# Aqueous humor neutrophil gelatinase-associated lipocalin levels in patients with idiopathic acute anterior uveitis

**Published:** 2010-07-29

**Authors:** David Salom, Empar Sanz-Marco, Jose L. Mullor, Maria Jesus Lopez-Prats, Salvador Garcia-Delpech, Patricia Udaondo, Jose Maria Millan, J. Fernando Arevalo, Manuel Diaz-Llopis

**Affiliations:** 1 Department of Ophthalmology, La Fe University Hospital of Valencia, Valencia, Spain; 2 Biomedical Network Research Centre on Rare Diseases (CIBERER), Valencia, Spain; 3 Department of Ophthalmology of the Valencia University, Valencia, Spain; 4 Instituto de Investigación Sanitaria, Fundación para la Investigación del Hospital La Fe, Valencia, Spain; 5 Experimental Ophthalmology Unit, Department of Ophthalmology, La Fe University Hospital of Valencia, Valencia, Spain; 6 Department of Ophthalmology of the Catholic University San Vicente Martir, Valencia, Spain; 7 Department of Genetics, La Fe University Hospital of Valencia, Valencia, Spain; 8 Clínica Oftalmológica Centro Caracas, Retina & Vitreous Service, Caracas, DC, Venezuela

## Abstract

**Purpose:**

The purpose of this study was to evaluate the levels of neutrophil gelatinase-associated lipocalin (NGAL) in the aqueous humor in eyes with idiopathic acute anterior uveitis (AAU).

**Methods:**

A comparative control study. Aqueous humor was collected from 20 eyes of 20 patients with idiopathic AAU. The control group included 20 aqueous samples from 20 patients about to undergo cataract surgery and without any other ocular or systemic diseases. The level of NGAL was determined with a commercially available ELISA kit.

**Results:**

The concentration of NGAL in aqueous humor was markedly higher in patients with idiopathic AAU than in control subjects (Mann–Whitney U test, p<0.001). The level of NGAL was 139,197.38±183,426.36 (mean±SD) pg/ml in eyes with AAU and 3,169.96±1,595.78 pg/ml in the eyes of the control group.

**Conclusions:**

The aqueous humor NGAL level is increased in eyes with idiopathic AAU. These results imply that NGAL is associated with the regulation of inflammation in patients with AAU and could be used as a biomarker of ocular inflammation and immunomodulatory treatment response.

## Introduction

The term “uveitis” is used to describe a group of diseases characterized by inflammation of intraocular structures. In the United States, uveitis is reportedly responsible for an estimated 30 000 new cases of legal blindness annually and causes 2.8% to 10% of cases of blindness [1,2], with a prevalence reported as high as 115,3 cases per 100,000 [2]. The non-infectious uveitis can be associated with autoimmune systemic diseases such as Behçet’s disease, sarcoidosis, spondyloarthropathies, and Vogt-Koyanagi-Harada syndrome, or unclassified uveitis are labeled idiopathic. Although posterior uveitis (affecting the posterior segment) describes a range of different clinical entities, all forms are similar immunohistologically, characterized by an infiltration of mainly lymphocytes T CD4+ (CD4^+^ T) cells [3].

Idiopathic acute anterior uveitis (AAU), in which there is often a severe inflammatory response in the anterior chamber, or front of the eye, is the most common type that occurs in the general population. The disease’s severity and course vary between individuals, and some patients have ocular complications that can threaten sight. Many different cytokines have been identified in the inflamed eye, including tumor necrosis factor (TNF)-α [4,5].

Neutrophil gelatinase-B associated lipocalin (Lcn2/NGAL or NGAL) is a 21-kD protein of the lipocalin superfamily. NGAL is siderophore-binding antimicrobial protein that is upregulated in epithelial tissues during inflammation and seems to play an important role in this process [6]. This protein is upregulated in several pathological conditions, including cancers [7], inflammation bowel disease [8], nephritis [9], acute kidney injury (AKI) [9], heart failure [10], autoimmune myocarditis [11], polyps [12], preeclampsia [13], arthritis [14], and pancreatitis [15]. Several studies have shown NGAL to be a useful biomarker for early detection of AKI in post-cardiac surgery, nephritis, and radiocontrast exposure [9].

The objective of this study was to measure the levels of NGAL in the aqueous humor of patients with idiopathic AAU and its possible implication as a regulator of inflammation in the acute phase of the uveitis.

## Methods

This comparative control study investigates the levels of NGAL in the aqueous humor of patients with idiopathic AAU. Control samples of aqueous humor were taken of cataract patients without other ocular or systemic disease. The study protocol complied with the Helsinki Declaration and was reviewed and approved by the Ethical Committee of our tertiary care hospital. An informed consent was obtained from each patient.

Aqueous humor samples were collected from 20 eyes of 20 patients with idiopathic AAU in their fist documented episode. A battery of serological and laboratory tests were performed in all patients to rule out any other ocular or systemic disease. Control aqueous humor samples were obtained from 20 eyes of 20 patients undergoing cataract surgery and without any other ocular or systemic confounding disease.

Aqueous samples of patients with AAU were collected under sterile conditions using a 30-gauge needle under the slit lamp and applying povidone-iodine before and after anterior chamber puncture. Topical antibiotic prophylaxis was used for 5 days after the sample was taken. The aqueous humor was collected from the controls with a 30-gauge needle before the start of cataract surgery. Samples were collected in volumes of at least 0.05 ml per patient, kept in sterile tubes, and stored immediately at −80 °C until use. NGAL levels were quantified by enzyme-linked immunosorbent assay (ELISA) of aqueous humor (Searchlight Array; Pierce Biotechnology, Inc., Woburn, MA). The demographic characteristics of patients were studied with the statistical program SPSS (SPSS Inc., Chicago, IL) for Windows. The Mann–Whitney U test for independent samples was applied to compare the levels of NGAL in the study groups, accepting p<0.05 as statistically significant.

## Results

We analyzed a total of 20 samples of aqueous humor of 20 patients with idiopathic AAU. The age of this group was 47±3.2 (mean±SD) years and 30% were women. Similarly we studied 20 samples of 20 patients that underwent cataract surgery, which constituted the control group, with an age of 55±2.7 (mean±SD) years, 40% were women.

**Figure 1:**
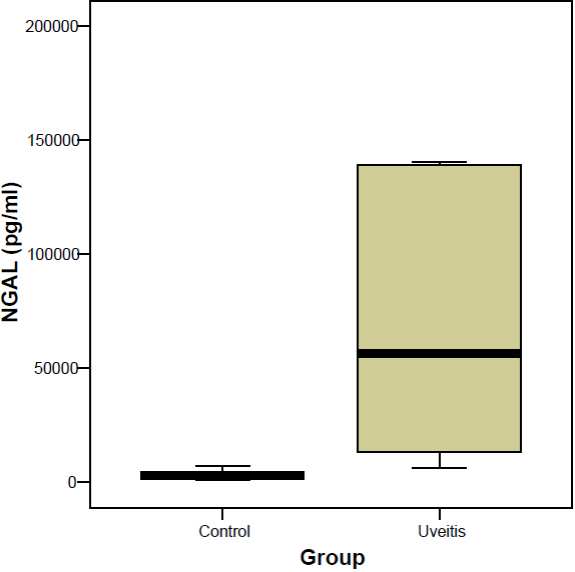
Levels of neutrophil gelatinase-associated lipocalin (NGAL) in aqueous humor of 20 eyes with idiopathic acute anterior uveitis (AAU) and 20 control eyes. NGAL levels were different in both groups; patients with AAU had significantly higher levels than in the control group (Mann–Whitney U test p<0.001).

The observed level of NGAL in eyes with idiopathic AAU was 139,197.38±183,426.36 pg/ml (mean±SD) and 3,169.96±1,595.78 pg/ml in the eyes of the control group (Table 1 and Table 2). The NGAL levels were significantly different between groups (Mann–Whitney U Test, p<0.001) in which AAU were significantly higher than that of the control group (Figure 1).

**Table 1:** Levels of neutrophil gelatinase-associated lipocalin (NGAL) in aqueous humor of all the patients.

	**Neutrophil gelatinase-associated lipocalin levels in aqueous humor (pg/ml)**	
**Patient**	**Acute anterior uveitis group**	**Control group**
1	12307.5	5030.2
2	500000	2157
3	124925	2565.3
4	433957.2	1264.3
5	500000	1365.5
6	81156.4	891.1
7	35275.6	3116.9
8	13484.5	7163.9
9	17411.9	5115.1
10	14422.5	1685
11	6610.3	1800
12	12704.3	2100
13	11474.8	2360
14	21760.2	4685
15	77611.4	4400
16	500000	4215
17	6253.8	4050
18	136100	3185
19	140294.8	3400
20	138197.4	2850
	139197.38±183426.36 pg/ml (mean±SD)*	3169.96±1595.78 pg/ml (mean±SD)*

The asterisk indicates a statistically significant difference between groups (Mann–Whitney U test; p<0.001)

**Table 2:** Results of the levels of neutrophil gelatinase-associated lipocalin (NGAL) in aqueous humor in each group.

**Group**	**Aqueous humor level of NGAL (pg/ml) mean ± SD**
AAU (n=20)	139197.38 ± 183426.36
	p<0.001*
Control (n=20)	3169.96 ± 1595.78

The group of patients with idiopathic acute anterior uveitis (AAU) had higher NGAL levels than the control group. NGAL=Neutrophil gelatinase-associated lipocalin; AAU=Acute Anterior Uveitis. *Compared with Control group (Mann–Whitney U test).

## Discussion

In our study, quantification of NGAL in aqueous humor was conducted in a homogeneous group of patients with idiopathic AAU in which we observed significantly elevated levels of NGAL compared with control subjects. These results imply that NGAL is associated with the regulation of inflammation in patients with AAU. In addition to our direct observation, NGAL is strongly upregulated by interleukin (IL) 1 beta [16] and TNF-α in the presence of IL-17 [17], a pro-inflammatory cytokine produced by the newly discovered subset of CD4+ T helper cells (TH-17), and increased levels of IL-1 has been observed in AAU [4], and this could be one of the reasons of the high levels found in our patients.

Otherwise, NGAL plays a cytoprotective role by transporting iron into cells, promoting differentiation in myocardial [11] and renal cells [18], developing an antioxidant activity and reducing apoptosis. NGAL is a potent inducer of heme oxygenase-1 (HO-1) and superoxide dismutase (1, 2; SOD) [19]. The overexpression of NGAL, significantly reduce cell death by apoptosis-inducing agents related to COX-2 and lipoxygenase inhibitors [19]. Moreover, NGAL is strongly induced by growth factors including insulin-like growth factor (IGF) and TGF-α in primary human keratinocytes, this induction is believed to play a role in wound healing [20]. This suggests that increased levels of NGAL may serve to limit injury in recurrent insults.

Plasma NGAL is a useful early preclinical marker for AKI in a heterogeneous adult intensive care unit population. In addition, it predicts need for renal replacement therapy and correlates with AKI severity [21]. Moreover, the levels of serum NGAL in patients with vasculitis, had a closer correlation with clinical findings (Birmingham Vasculitis Activity Score, BVAS) than erythrocyte sedimentation rate, C-reactive protein, and anti-neutrophil cytoplasmic antibody did [22]. Other studies have confirmed the potential usefulness of NGAL measurement in the evaluation of early responses to therapy or in predicting different clinical outcomes with infliximab in Crohn disease [6] and intravenous immunoglobulin infusion in renal disease [23]. However, the serum levels of NGAL in uveitis have not been determined yet. It would be interesting to analyze the correlation of NGAL serum levels with aqueous humor levels to determine whether NGAL can be used as a biomarker of inflammatory activity. In this context, NGAL could be used to molecularly quantify and monitor the response to an anti-inflammatory treatment, not only in AAU, but also, in inflammatory diseases of different origins.

In summary, the aqueous humor NGAL levels are increased in eyes with idiopathic AAU. We suggest NGAL is involved in the inflammatory response in AAU where it may act as a cytoprotective factor. As was previously reported, NGAL was associated with high levels of gelatinase B in patients with active uveitis, suggesting that neutrophils is a significant source of gelatinase B in these patients. Gelatinase B is a specifically cleaver of type IV collagen of endothelial basement membrane, and may be responsible of the blood-ocular barrier disruption in patients with uveitis [24]. Our results add evidence to the possibility of using selective gelatinase inhibitors as a potential therapy in patients with uveitis. The aqueous humor levels of NGAL may be used as a biomarker of inflammatory ocular activity in patients with idiopathic AAU.
